# Correction: Interferon receptor-deficient mice are susceptible to eschar-associated rickettsiosis

**DOI:** 10.7554/eLife.110283

**Published:** 2025-12-19

**Authors:** Thomas P Burke, Patrik Engström, Cuong J Tran, Ingeborg M Langohr, Dustin R Glasner, Diego A Espinosa, Eva Harris, Matthew D Welch

**Keywords:** Mouse

Burke TP, Engström P, Tran CJ, Langohr IM, Glasner DR, Espinosa DA, Harris E, Welch MD. 2021. Interferon receptor-deficient mice are susceptible to eschar-associated rickettsiosis. *eLife* **10**:e67029. doi: 10.7554/eLife.67029.

Published 23 August 2021

After publication, we became aware that due to an oversight we incorrectly wrote in the Results that Fig. 4i was *Ifnar*^-/-^*Ifngr*^-/-^mice, when in fact it was wild type (WT) mice. The legend for Fig. 4i correctly stated that it is WT mice. This doesn't affect any conclusions for the paper, and no new data need to be uploaded or changed.

Correct text in the Results:

"However, when we examined bacterial burdens upon i.v. infection of WT mice with *R. parkeri*, similar amounts of WT and *sca2*::Tn bacteria were recovered in spleens (Figure 4i). We were also surprised to find that significantly more *sca2*::Tn than WT *R. parkeri* were recovered in livers (Figure 4i)."

Original published text in the Results:

"However, when we examined bacterial burdens upon i.v. infection of *Ifnar1*-/-;*Ifngr1*-/- mice with *R. parkeri*, similar amounts of WT and *sca2*::Tn bacteria were recovered in spleens (Figure 4i). We were also surprised to find that significantly more *sca2*::Tn than WT *R. parkeri* were recovered in livers (Figure 4i)."

The article has been corrected accordingly.

